# No Surprises Act Independent Dispute Resolution Outcomes for Air Ambulances

**DOI:** 10.1001/jamanetworkopen.2024.62404

**Published:** 2025-03-03

**Authors:** Erin L. Duffy, Christopher Garmon

**Affiliations:** 1 Schaeffer Center for Health Policy & Economics, University of Southern California, Los Angeles; 2 Department of Public Affairs, Henry W. Bloch School of Management, University of Missouri–Kansas City, Kansas City

## Abstract

**Importance:**

The No Surprises Act (NSA) banned surprise patient balance bills and established a binding arbitration system to resolve payment disputes between insurers and health care providers (eg, clinicians, hospitals, air ambulance organizations) for certain services, including air ambulance transportation. Understanding use and results of this new independent dispute resolution (IDR) system can inform ongoing adjustments to its implementation.

**Objective:**

To describe the involved parties and outcomes of air ambulance NSA IDR cases.

**Design, Setting, and Participants:**

This cross-sectional study assessed cases arbitrated through NSA IDR in 2023 as reported by the Centers for Medicare & Medicaid Services. Participants included air ambulance organizations and commercial insurers. Data were analyzed between August 1 and September 1, 2024.

**Exposures:**

Fixed-wing and rotary transport disputes arbitrated under the NSA IDR system.

**Main Outcomes and Measures:**

The activation components of fixed-wing and rotary transport disputes were evaluated with data from 2 distinct public files: (1) disclosed parties and outcomes relative to qualifying payment amounts (QPAs), the insurers’ median of contracted rates for similar services in the region among similar health plans; and (2) monetary measures but without parties. The first file describes the insurers and organizations involved, including their ownership status, as well as the prevailing party and the offers and decisions relative to QPA. The second file describes QPAs, offers, and decisions monetarily and these are compared with Medicare’s urban allowed amount.

**Results:**

There were 5678 IDR cases in 2023, comprising disputes for 4935 rotary and 743 fixed-wing transports. Air ambulance organizations offered a mean (SD) of 3.18 (4.20) times QPA, and insurers offered 1.39 (3.89) times QPA. Organizations won 4905 cases (86.4%) at a mean (SD) of 2.95 (4.12) times the QPA. A total of 3478 cases (61.3%) involved organizations owned by private equity investors, and these cases had higher prevailing offers relative to the QPA. Due to data suppression, analysis of financial measures was possible for 2897 of 5983 activation cases (48.4%), among which the mean (SD) QPA offer was $15 561.08 ($9730.97) (3.74 [2.31] times Medicare), and the winning offer was $32 463.70 ($9987.17) (7.82 [2.39] times Medicare).

**Conclusions and Relevance:**

In this cross-sectional study of air ambulance payment disputes, air ambulance organizations won most cases, requiring insurers to pay more in these cases. Notably, the NSA does not require changes to patient cost-sharing based on IDR outcomes. Assessment of the financial metrics and outcomes of air ambulance cases was limited due to missing and masked information. Continued monitoring of air ambulance IDR cases is needed to assess the impacts of the NSA.

## Key Points

**Question:**

What were the outcomes of air ambulance cases processed by the No Surprises Act’s independent dispute resolution entities in 2023?

**Findings:**

In this cross-sectional study of 5678 cases, arbiters decided in favor of air ambulance organizations in 86.4% of the disputes against insurers, and 61.3% of cases involved a private equity–backed organization. Financial measures were reported for only 48.4% cases; among them, the mean qualifying payment amount (a measure of the insurer’s median contracted rate) and winning offers were $15 561 and $32 463, respectively.

**Meaning:**

These findings suggest that more complete public data on air ambulance independent dispute resolution cases is needed to monitor the impacts of the No Surprises Act.

## Teaser

This cross-sectional study describes independent dispute resolution cases arbitrated in 2023 for fixed and rotary wing air ambulance services following the No Surprises Act.

## Introduction

Air ambulance transportation comprises travel by fixed-wing and rotary (helicopter) aircraft and is used to transport patients from scenes of an emergency or between facilities when time or travel conditions prohibit use of a ground ambulance. These services are used annually by 1 in 4000 commercially insured people and 1 in 350 Medicare beneficiaries.^1^ Patients do not typically choose the air transportation organization serving them due to the nature of emergencies, geography, and logistics involved. Air transport availability for emergency scenarios requires pilots, clinicians, and aircraft to be prepared for flight at all times. Air ambulance organizations make significant investment in infrastructure, maintenance, and staff to maintain readiness and comply with Federal Aviation Administration regulations, and they face uncertain expected utilization.

Prior to the implementation of the No Surprises Act (NSA) in 2022, commercially insured patients could be billed for the balance between an out-of-network air transportation organization’s charges and their insurer’s allowed amount (ie, a surprise bill), while Medicare and Medicaid patients were insulated from such bills. A study of commercial insurance claims from 2013 to 2017 found that most air ambulance services were out-of-network, and more than half left patients vulnerable to potential surprise bills averaging approximately $20 000.^2^ Other studies evaluating air ambulance charges found they were highly variable, with higher charges among larger organizations and organizations that are publicly traded and have private equity (PE) ownership.^3,4^ PE-owned air ambulance organizations have been found to receive higher payments and yield more and larger surprise bills than organizations with hospital, nonprofit, and independent ownership arrangements.^5^

The NSA was passed in December 2020 and implemented in January 2022. This federal law banned surprise billing for air ambulance transport as well as for emergency care and out-of-network services at an in-network facility. The NSA also established the independent dispute resolution (IDR) process to resolve payment disagreements between health care providers (eg, physicians, hospitals, air ambulance organizations) and insurers. In this binding final-offer arbitration system, arbiters must choose either the insurer’s offer or the provider’s offer, taking into consideration several factors, including any past contracts between the parties, the details of the service conducted, and the insurer’s computed median contracted rate for the same or similar service in the given market, which is known as the qualifying payment amount (QPA).

The implementation of the IDR has been punctuated by litigation that has altered rulemaking and triggered closure of the IDR portal from August through December of 2023.^6,7^ Specific to air ambulance services, the *LifeNet, Inc, et al v US Department of Health and Human Services et al *case challenges QPA calculation methodology.^8^ Additionally, ongoing cases *Guardian Flight, LLC, v Medical Evaluators of Texas* in the Fifth Circuit^9^ and *REACH Air Medical Services LLC v Kaiser Foundation Health Plan Inc et al *in the Eleventh Circuit^10^ both challenge the validity of IDR entities’ arbitration decisions.

Monitoring and analyzing the outcomes of the IDR process can inform evolving rulemaking and shed light on the fiscal impacts of the NSA. This study describes IDR cases arbitrated in 2023 for fixed-wing and rotary air ambulance services. Though there have been other studies of IDR outcomes, to our knowledge this is the first study focusing on air ambulance services.^11,12,13^

## Methods

### Data Source

This cross-sectional study used public IDR reports published by the Centers for Medicare & Medicaid Services (CMS) for each quarter of 2023.^14^ Although the portal was closed to new IDR cases for part of 2023, the backlog of previously submitted cases continued to be arbitrated during this period. Every air transport generates 2 claims: one for activation (akin to a base fee for the flight) and one for mileage. In the IDR system, activation and mileage services are reported as separate disputes and are unpaired. Mileage disputes do not report the miles traveled in the data released by CMS, limiting the interpretability of these dispute outcomes. With these data limitations, this study focused only on activation disputes for rotary (*Current Procedural Terminology* [*CPT*] code A0431) and fixed-wing (*CPT* code A0430) transports. The institutional review board of the University of Southern California, Los Angeles, determined this study was not human participant research. This report follows the Strengthening the Reporting of Observational Studies in Epidemiology (STROBE) reporting guideline.

Data are provided by CMS in 2 files that cannot be merged, each with strengths and limitations. This data structure was presumably designed by CMS to deidentify insurers’ QPA amounts. The first file contains observations of each air ambulance dispute with disclosed parties and outcomes relative to QPA (nonmonetary file). The advantage of this file is that it enables one to observe which air ambulance organizations and insurers are disputing cases against one another. A limitation of this file is that it expresses magnitudes of offers and decisions as ratios to QPA values, without disclosing the underlying QPA values in dollars. Given that there is variation in QPA values, these ratios cannot be used to reliably interpret the financial magnitude of offers and decisions. The second file contains observations of disputes with monetary measures of offers and decisions without identifying parties involved (monetary file). The advantage of this file is that it discloses dollar values, enabling benchmarking to Medicare and consideration of the financial implication of the NSA. However, this file has missing data for many observations, and it does not disclose the parties involved in disputes. We leverage the strengths of each file to gain insights on the parties involved and outcomes of IDR cases.

### Measures

The number of activation components of fixed-wing and rotary transport disputes, the parties involved, and the prevailing party were described using the nonmonetary file. The air ambulance organization offer, insurer offer, and arbiter decisions were reported relative to the QPA. These case attributes were described separately for organizations with and without PE investments (as identified in eTable 1 in Supplement 1), as well as by quarter of the year. Density maps were used to describe the count of disputes from each state.

The QPA, air ambulance organization offer, insurer offer, and arbiter decisions were described in nominal 2023 US dollars using the monetary file. Monetary values were also compared with Medicare urban reimbursement rates. Rural or urban pickup designations are an important element in air ambulance billing, but geographic information in the data released by CMS was not sufficient to determine an urban or a rural pickup location. For comparison with Medicare, we apply the urban pickup rate in the primary analysis.

### Statistical Analysis

Data were analyzed between August 1 and September 1, 2024. Categorical outcomes are described with frequencies and percentages. Continuous outcomes are described with means and SDs. Histograms are also used to assess the distributions of prevailing offers and QPA values. Three ratios of winning value to QPA that exceeded 1000 were excluded as outliers, with the next highest value being 184, strongly suggesting that these 3 outliers are erroneous entries. Three observations with a negative winning value to QPA ratio were also excluded. All statistical analyses were performed using Stata, version 18 (StataCorp LLC). For analyses that combine rotary and fixed-wing services, the descriptive statistics for each wing type are included separately in eTables 2, 3, 5, and 6 and eFigure 2 in Supplement 1.

## Results

**Figure 1.**
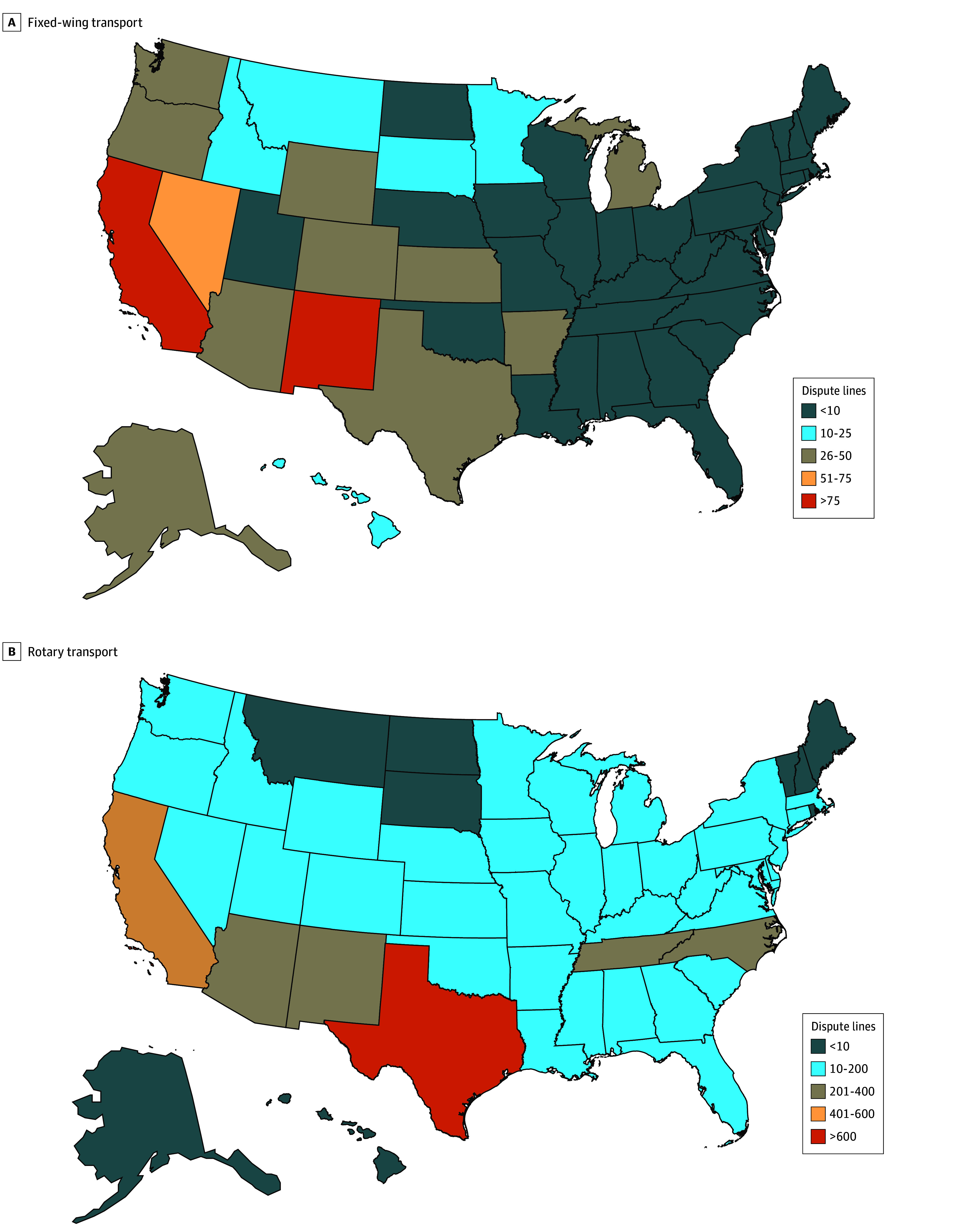
Maps of Dispute Case Counts by State Negative winning values and outliers are excluded.

The analytic sample from the nonmonetary file included 743 fixed-wing and 4935 rotary transports for a total of 5678 disputes (Table 1). Air ambulance organizations won 86.4% of the cases (4905 of 5678) at a mean (SD) magnitude 2.95 (4.12) times the QPA, applying ratios to QPA reported in the nonmonetary file. Plans offered a mean (SD) of 1.39 (3.89) times the QPA, and air ambulance organizations offered 3.18 (4.20) times the QPA. The distributions of ratios of winning offers to QPA are shown in eFigures 1 and 2 in Supplement 1. In 52.6% of cases (2986 of 5678), the health plan offered more than the QPA in arbitration. Most of the 5678 disputes involved the organizations Global Medical Response (2414 [42.5%]), Phi Air Medical (1334 [23.5%]), and Air Methods Corporation (791 [13.9%]). Health plan participation in IDR was spread among many insurance carriers, as well as insurer partners MultiPlan Corporation and Zelis. Rotary transport disputes came from states all across the country, while fixed-wing transport disputes were concentrated in some Western states, particularly California, New Mexico, and Nevada (Figure 1).

**Table 1.** Characteristics of Disputes for Fixed-Wing and Rotary Transport Activation^a^

Characteristic	No. (%) of dispute lines		
	Fixed wing (n = 743)	Rotary wing (n = 4935)	All (N = 5678)
Health plan offer >QPA			
No	331 (44.5)	2361 (47.8)	2692 (47.4)
Yes	412 (55.5)	2574 (52.2)	2986 (52.6)
Outcome			
Health plan wins	125 (16.8)	644 (13.0)	769 (13.5)
Air ambulance organization wins	618 (83.2)	4287 (86.9)	4905 (86.4)
Split decision	0	4 (0.1)	4 (0.1)
Air ambulance parent organization			
Global Medical Response (PE)	402 (54.1)	2012 (40.8)	2414 (42.5)
Phi Air Medical (PT)	10 (1.3)	1324 (26.8)	1334 (23.5)
Air Methods (PE)	72 (9.7)	719 (14.6)	791 (13.9)
Apollo MedFlight (PE)	50 (6.7)	223 (4.5)	273 (4.8)
Life Flight	51 (6.9)	144 (2.9)	195 (3.4)
Other	158 (21.3)	513 (10.4)	671 (11.8)
Health plan parent organization			
Aetna Inc (PT)	72 (9.7)	602 (12.2)	674 (11.9)
Anthem Inc (PT)	88 (11.8)	279 (5.7)	367 (6.5)
Non-Anthem Blue	247 (33.2)	1209 (24.5)	1456 (25.6)
Centene Corporation (PT)	6 (0.8)	189 (3.8)	195 (3.4)
Cigna Group (PT)	27 (3.6)	227 (4.6)	254 (4.5)
Clear Health Strategies LLC^b^	52 (7.0)	308 (6.2)	360 (6.3)
Kaiser Permanente	17 (2.3)	280 (5.7)	297 (5.2)
MultiPlan Corporation (PT)	61 (8.2)	528 (10.7)	589 (10.4)
UHC (PT)	25 (3.4)	449 (9.1)	474 (8.3)
Zelis (PE)	30 (4.0)	239 (4.8)	269 (4.7)
Other	118 (15.9)	625 (12.7)	743 (13.1)
Air ambulance organization offer as multiple of QPA, mean (SD)	3.74 (3.00)	3.09 (4.34)	3.18 (4.20)
Health plan offer as multiple of QPA, mean (SD)	1.31 (0.83)	1.40 (4.16)	1.39 (3.89)
Winning offer as multiple of QPA, mean (SD)	3.33 (2.87)	2.89 (4.27)	2.95 (4.12)

Abbreviations: PE, private equity; PT, publicly traded; QPA, qualifying payment amount.

^a^ Data are from the nonmonetary file, excluding negative winning values and outliers.

^b^ Formerly PE.

PE-backed air ambulance organizations represented 61.3% of dispute lines (3478 of 5678) (Table 2). Organizations with and without PE backing won a similar proportion of cases, at 86.9% (3023 of 3478) and 85.5% (1882 of 2200), respectively. Mean (SD) winning offers for PE organizations were 3.52 (4.94) times the QPA, while winning offers for non-PE organizations were 2.04 (1.96) times the QPA. The mean insurer offer and organization offer relative to QPA were greater when the dispute involved a PE-backed organization compared with when the organization was not PE backed. These results are shown disaggregated by fixed-wing and rotary transport in eTables 2 and 3 in Supplement 1, and the disaggregated results are similar to the aggregate results.

**Table 2.** Characteristics of Disputes for Air Transport Activation by Organization PE Investment Status^a^

Characteristic	Dispute lines, No. (%)		
	Non-PE (n = 2200)	PE (n = 3478)	Total (N = 5678)
Health plan offer >QPA			
No	1190 (54.1)	1502 (43.2)	2692 (47.4)
Yes	1010 (45.9)	1976 (56.8)	2986 (52.6)
Outcome			
Health plan wins	314 (14.3)	455 (13.1)	769 (13.5)
Air ambulance organization wins	1882 (85.5)	3023 (86.9)	4905 (86.4)
Split decision	4 (0.2)	0	4 (0.1)
Air ambulance organization offer as multiple of QPA, mean (SD)	2.18 (1.99)	3.80 (5.02)	3.18 (4.20)
Health plan offer as multiple of QPA, mean (SD)	1.12 (1.01)	1.58 (4.96)	1.39 (3.89)
Winning offer as multiple of QPA, mean (SD)	2.04 (1.96)	3.52 (4.94)	2.95 (4.12)

Abbreviations: PE, private equity; QPA, qualifying payment amount.

^a^ Analysis of nonmonetary file excludes negative winning values and outliers. Analysis includes both fixed-wing and rotary transports.

Across the 4 quarters of 2023, we observed an increase in the share of disputes won by health plans from 9.1% (114 of 1250) in quarter 1 to 17.1% (200 of 1170) in quarter 4 (eTable 4 in Supplement 1). Accompanying this trend, we observed that health plans increasingly made offers above the QPA—33.8% of the time (422 of 1250 cases) in quarter 1 and 59.1% of the time (692 of 1170 cases) in quarter 4. The mean (SD) number of days for the arbiters to make decisions also extended over the year from 55 (25) days in quarter 1 to 136 (68) days in quarter 4, reflecting the backlog of cases and the pausing of new cases in the latter part of the calendar year. Quarterly results are shown disaggregated by fixed-wing and rotary transport in eTables 5 and 6 in Supplement 1, and the disaggregated results are similar to the aggregate results.

**Figure 2.**
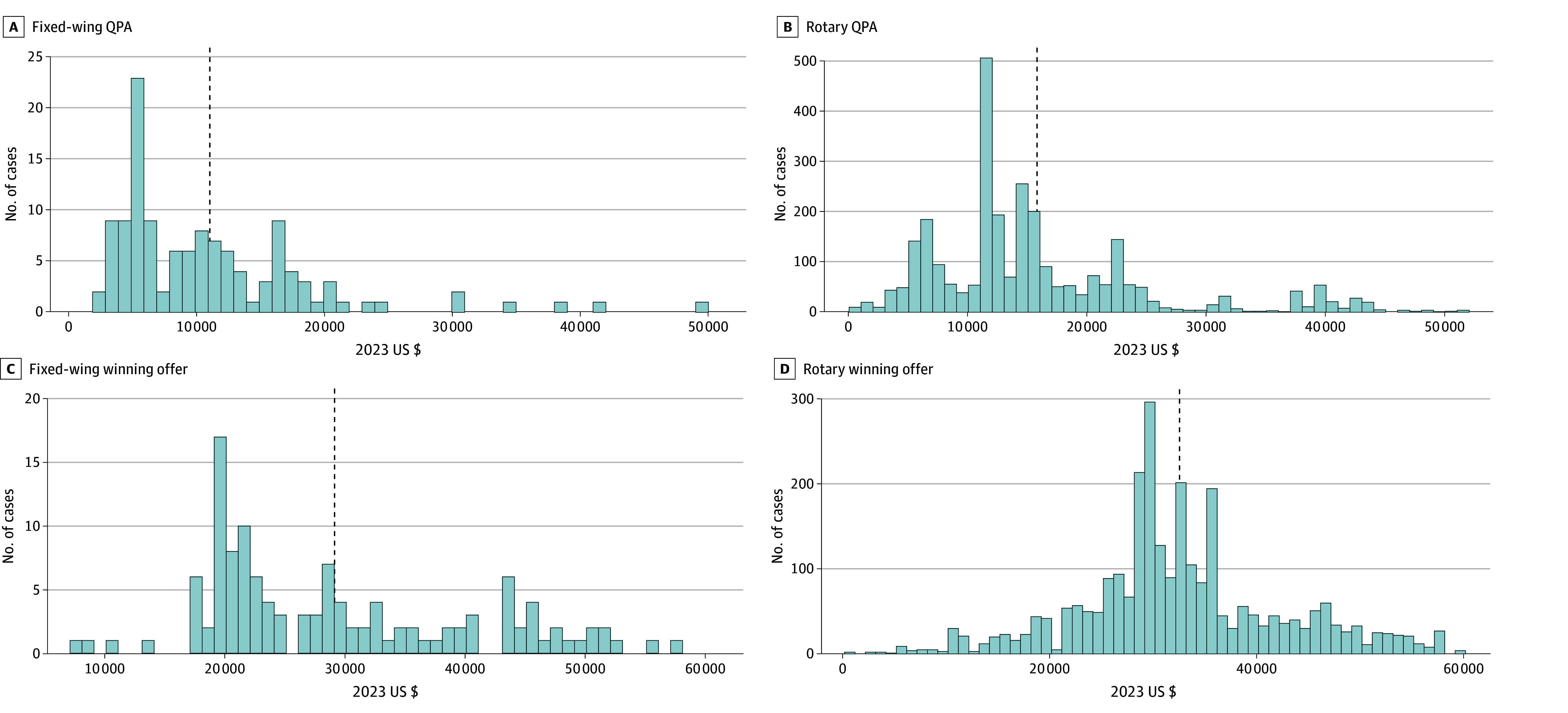
Histograms of Qualifying Payment Amounts (QPA) and Winning Offers for Rotary and Fixed-Wing Transport Initiations The vertical dashed lines represent the means. Mean (SD) QPA and winning offers are given in Table 3.

The monetary analytic file includes 126 fixed-wing and 2929 rotary transport disputes, totaling 3055 cases (Table 3). Notably, this is much lower than the 5678 cases reported in the nonmonetary file. The mean QPA and the air ambulance organization, health plan, and prevailing offers for rotary activation disputes were greater than for fixed-wing activation disputes. Overall, mean (SD) QPA was $15 561.08 ($9730.97) and prevailing offers were for a mean (SD) of $32 463.70 ($9987.17). The mean (SD) air ambulance organization and health plan offers were $34 290.54 ($8985.03) and $16 742.90 ($15 088.73), respectively. QPAs and prevailing offers were a mean (SD) of 3.74 (2.31) and 7.82 (2.39) times the Medicare urban allowed amounts, respectively. Figure 2 presents the distributions of QPA and prevailing offers for rotary and fixed-wing transport disputes, demonstrating the substantial variation in these values.

**Table 3.** Monetary Dispute Offers by Fixed-Wing and Rotary Transports^a^

Variable	Transport, Mean (SD)		
	Fixed-wing (n = 126)	Rotary (n = 2929)	All (n = 3055)
QPA, US $	11 080.62 (8078.10)	15 755.40 (9751.05)	15 561.08 (9730.97)
Offer, US $			
Air ambulance organization	29 676.08 (11 326.36)	34 499.24 (8810.12)	34 290.54 (8985.03)
Health plan	9038.34 (5323.46)	16 788.00 (15 116.26)	16 742.90 (15 088.73)
Prevailing	29 115.42 (11 292.36)	32 610.97 (9902.10)	32 463.70 (9987.17)
Ratio of QPA to Medicare urban rate, mean (SD)	3.09 (2.24)	3.77 (2.31)	3.74 (2.31)
Ratio of offer to Medicare urban rate, mean (SD)			
Air ambulance organization	8.32 (3.22)	8.26 (2.07)	8.26 (2.14)
Health plan	2.70 (1.59)	4.03 (3.75)	4.02 (3.74)
Prevailing	8.15 (3.20)	7.81 (2.35)	7.82 (2.39)

Abbreviation: QPA, qualifying payment amount.

^a^ Analysis of monetary file is reported in 2023 nominal US dollars.

Monetary aspects of IDR cases were masked at a far higher rate for air ambulance transport than other services (eTable 7 in Supplement 1). For air ambulance activation dispute lines, 48.4% of dispute lines (2897 of 5983) report the monetary value of both the QPA and winning offer. Thus, analysis of the monetary aspects of disputes was not feasible for more than half of air ambulance disputes. In contrast, 85.0% of emergency services (140 232 of 165 024) and 64.7% of other services (108 999 of 168 481) report both the QPA and winning offer in dollars in the publicly released files.

## Discussion

In this cross-sectional study of 2023 NSA IDR outcomes for air ambulance transport disputes, air ambulance organizations won 86.4% of cases at mean (SD) amounts of 2.95 (4.12) times the QPA and 7.82 (2.39) times Medicare reimbursements. These are higher than pre-NSA in-network allowed amounts, which were 3 to 5 times Medicare reimbursements,^15,16^ but roughly consistent with pre-NSA charges, which were 4 to 9 times Medicare reimbursements.^3^

This study also found that PE-backed air ambulance organizations comprised more than half (61.3%) of dispute lines and yielded higher prevailing amounts in the IDR process than organizations without PE investment. A study of 2016-2017 commercial claims data^5^ reported that PE and publicly traded air ambulance organizations had higher allowed amounts for both in-network and out-of-network services than independent, hospital-owned, and nonprofit air ambulance organizations. This same pre-NSA study also reported that PE and publicly traded air ambulances were more often out-of-network and more often generated possible surprise billing scenarios. Air ambulance companies can no longer bill for the balance, but the higher IDR winnings for PE-backed organizations observed in this study suggest that the payment advantage of PE-backed organizations may have persisted after the NSA. The relatively frequent use of IDR by large PE-backed and publicly traded organizations may indicate that the costs and administrative processes of the IDR system are discouraging its use by smaller independent and nonprofit organizations. Composing a compelling case for arbitration may require time and effort from clinicians, drawing them away from treating patients, and this use of expertise may not be feasible for smaller organizations.

At the time of this analysis, only 2023 IDR data were publicly available to researchers, released in quarterly files. The quarter assignments in these data are based on the quarter an arbiter decided the case. There was an increase in the share of IDR cases with insurer offers above the QPA and an increase in the proportion of cases that insurers won during the 4 quarters of 2023. This suggests that insurers may have adapted their strategy in IDR offers over time, perhaps based on their experience with IDR. Important context for interpreting this trend is that the IDR was closed to new submission from August through December 2023 and the system experienced a backlog and processing time lag.^7^ Therefore, the offers we saw in quarters 3 and 4 were actually made much earlier. We saw a snapshot of IDR activity, and the offers and decisions may have changed in the months since then.

The future of the IDR process depends on the outcomes of several ongoing legal cases about the QPA calculation methodology and the validity of arbitration decisions.^8,9,10^ Additionally, the first reported audit of an insurance carrier’s QPA calculation was released in July 2024.^17^ The audit focused on Aetna Inc’s handling of air ambulance cases and identified 3 violations, including the use of paid claims rather than contracts in QPA calculations. The resolution of NSA-related litigation and further auditing of QPA calculations may change future IDR outcomes.

Monitoring of the IDR outcomes for air ambulance services is constrained by the limited public data for these cases. The separation of activation and mileage claims for the same flight makes it difficult to assess the overall IDR payment outcome for a transport. The lack of mileage and information on pickup location limits the accuracy of benchmarking payments. CMS could make more complete data available for monitoring and consider grouping the activation and mileage components of air transport claims as disputes are processed. This transparency would inform the behavior of insurers and air ambulance organizations, and the clinicians involved, as they engage in the IDR process.

### Limitations

The publicly available IDR data have several limitations. Though air ambulance transportation services always comprise paired activation and mileage claims, these claims are not linked in the IDR data. The data do not include mileage or pickup location designation as rural or urban, limiting interpretability and benchmarking accuracy. The file with detailed party information cannot be linked with monetary information, making it infeasible to assess variation in offers and win amounts by party. Nearly half of cases have masked data fields, and it is not possible to determine whether observations about the unmasked cases are generalizable to the masked cases.

This study captures a single year of IDR outcomes, 2023. These are the only IDR data available to the public as of September 1, 2024. Litigation and rulemaking are ongoing, so the observations in the study offer insights to inform that process. Future data releases can be analyzed to build on this study and understand the evolving landscape.

## Conclusions

In this cross-sectional study of payment disputes, air ambulance organizations won most disputes against health plans. PE-backed organizations were involved in 3 in 5 disputes, and their cases had higher organization, health plan, and prevailing monetary offers. Continued monitoring of air ambulance IDR cases is needed to assess the impacts of the NSA, particularly in light of ongoing litigation and opportunities to inform evolving rulemaking. However, due to suppression of observations in the monetary file and lack of mileage and urban or rural service information, it was not possible to get a comprehensive picture of the financial aspects of air ambulance transport IDR cases in the publicly released data.

## Notes

Supplement 1. **eTable 1.** Air Ambulance Organization Ownership Types **eFigure 1.** Histogram of Winning Offer to QPA (Nonmonetary File) **eFigure 2.** Histograms of Winning Offer to QPA Ratio by Wing Type and Winning Party (Nonmonetary File) **eTable 2.** Fixed Wing Activation Dispute Means and Counts by PE and Non-PE (Nonmonetary File) **eTable 3.** Rotary Activation Dispute Means and Counts by PE and Non-PE (Nonmonetary File) **eTable 4.** Characteristics of Disputes for Air Transport Activation by Quarter of 2023 (Nonmonetary File) **eTable 5.** Fixed Wing Activation Dispute Means and Counts by Quarter (Nonmonetary File) **eTable 6.** Rotary Activation Dispute Means and Counts by Quarter (Nonmonetary File) **eTable 7.** Masking by Type of Service (Monetary File)

Supplement 2.
Data Sharing Statement
