# Supplementary material for: No Surprises Act Independent Dispute Resolution Outcomes for Air Ambulances
Source: JAMA Netw Open. 2025 Mar 3;8(3):e2462404. doi: 10.1001/jamanetworkopen.2024.62404 (PMC11877184; doi:10.1001/jamanetworkopen.2024.62404)
Supplement: Supplement 1. — eTable 1. Air Ambulance Organization Ownership Types eFigure 1. Histogram of Winning Offer to QPA (Nonmonetary File) eFigure 2. Histograms of Winning Offer to QPA Ratio by Wing Type and Winning Party (Nonmonetary File) eTable 2. Fixed Wing Activation Dispute Means and Counts by PE and Non-PE (Nonmonetary File) eTable 3. Rotary Activation Dispute Means and Counts by PE and Non-PE (Nonmonetary File) eTable 4. Characteristics of Disputes for Air Transport Activation by Quarter of 2023 (Nonmonetary File) eTable 5. Fixed Wing Activation Dispute Means and Counts by Quarter (Nonmonetary File) eTable 6. Rotary Activation Dispute Means and Counts by Quarter (Nonmonetary File) eTable 7. Masking by Type of Service (Monetary File) [file jamanetwopen-e2462404-s001.pdf]

Supplementary Online Content

Duffy EL, Garmon C. No Surprises Act independent dispute resolution outcomes for air ambulances. *JAMA Netw Open*. 2025;8(3):e2462404. doi:10.1001/jamanetworkopen.2024.62404

eTable 1. Air Ambulance Organization Ownership Types

eFigure 1. Histogram of Winning Offer to QPA (Nonmonetary File)

eFigure 2. Histograms of Winning Offer to QPA Ratio by Wing Type and Winning Party (Nonmonetary File)

eTable 2. Fixed Wing Activation Dispute Means and Counts by PE and Non-PE (Nonmonetary File)

eTable 3. Rotary Activation Dispute Means and Counts by PE and Non-PE (Nonmonetary File)

eTable 4. Characteristics of Disputes for Air Transport Activation by Quarter of 2023 (Nonmonetary File)

eTable 5. Fixed Wing Activation Dispute Means and Counts by Quarter (Nonmonetary File)

eTable 6. Rotary Activation Dispute Means and Counts by Quarter (Nonmonetary File)

eTable 7. Masking by Type of Service (Monetary File)

This supplementary material has been provided by the authors to give readers additional information about their work.

© 2025 Duffy EL and Garmon C. *JAMA Network Open*.

eTable 1. Air Ambulance Organization Ownership Types

Company	Type	Notes
**Global Medical Response**	Private Equity	
**Phi Air Medical**	Publicly-Traded	Division of Phi Group, Inc (OTCMKTS: PHIL)
**Air Methods**	Private Equity	Bankruptcy in October 2023
**Apollo MedFlight**	Private Equity	
**Life Flight**	Non-Profit	

© 2025 Duffy EL and Garmon C. *JAMA Network Open*.

eFigure 1. Histogram of Winning Offer to QPA (Nonmonetary File)

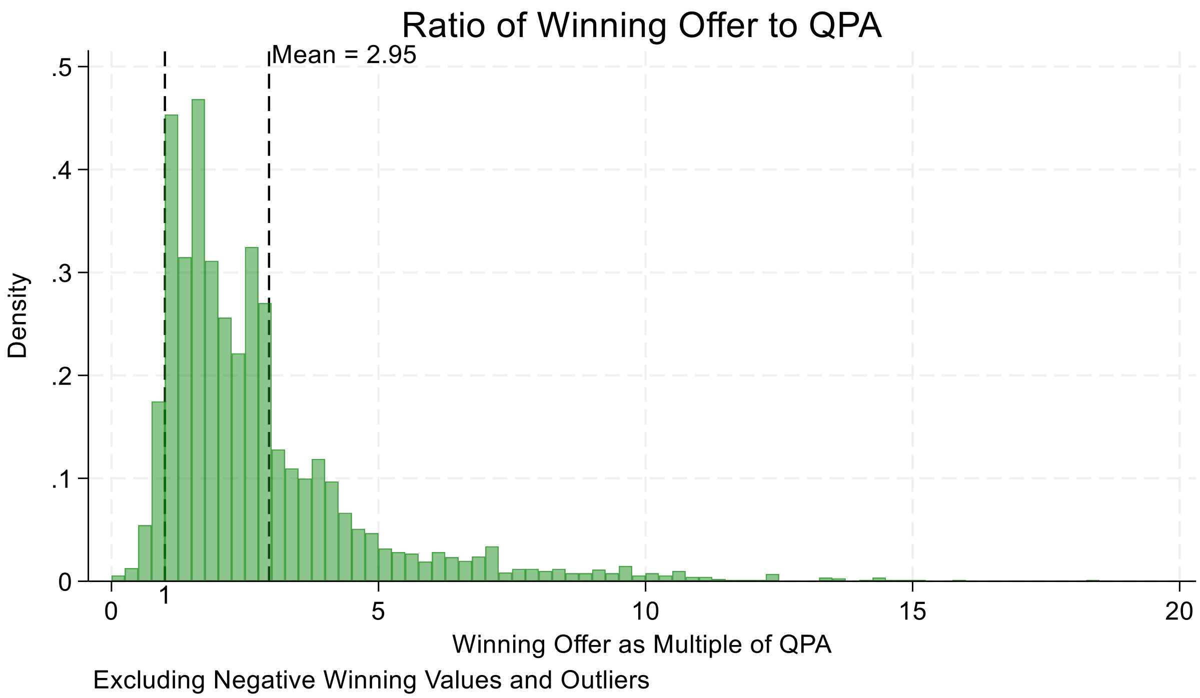

© 2025 Duffy EL and Garmon C. *JAMA Network Open*.

eFigure 2. Histograms of Winning Offer to QPA Ratio by Wing Type and Winning Party (Nonmonetary File)

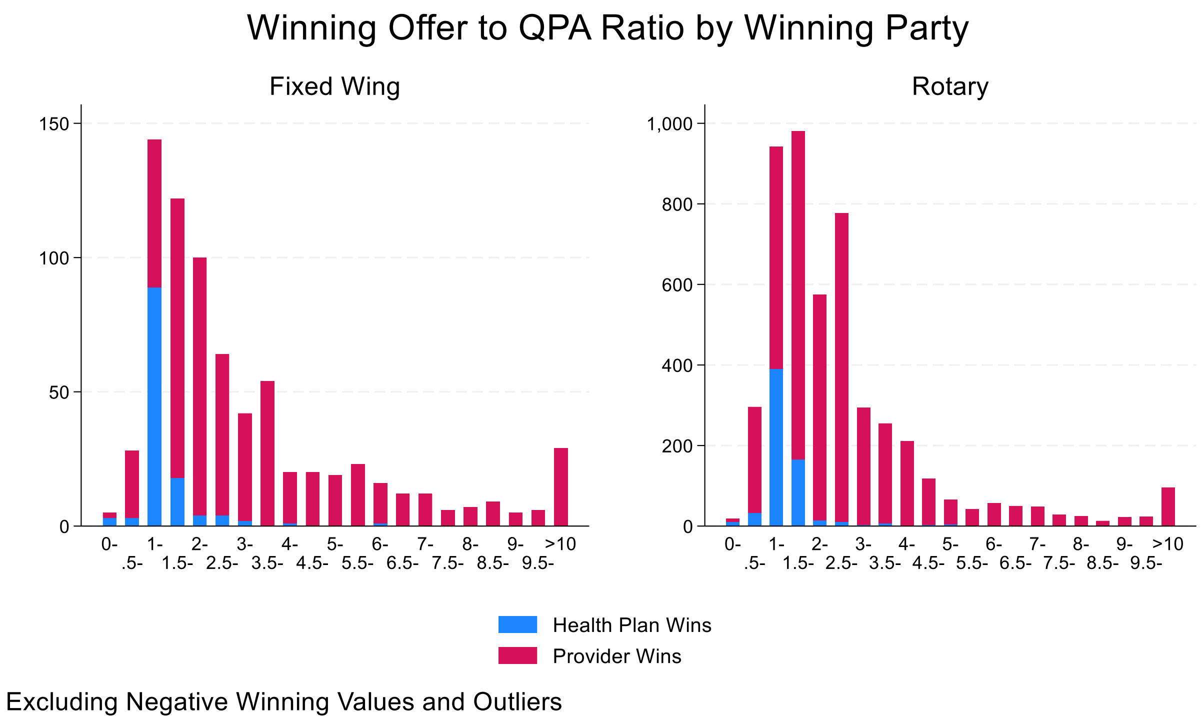

© 2025 Duffy EL and Garmon C. *JAMA Network Open*.

eTable 2. Fixed Wing Activation Dispute Means and Counts by PE and Non-PE (Nonmonetary File)

	Non-PE		Private Equity PE		Total	
	n	(%)	n	(%)	n	(%)
Dispute Lines	219		524		743	
Health PLan Offer > QPA						
No	99	45.2%	232	44.3%	331	44.5%
Yes	120	54.8%	292	55.7%	412	55.5%
Outcome						
Health Plan Wins	52	23.7%	73	13.9%	125	16.8%
Provider Wins	167	76.3%	451	86.1%	618	83.2%
	mean	(SD)	mean	(SD)	mean	(SD)
Provider Offer as Multiple of QPA	2.39	(1.37)	4.31	(3.30)	3.74	(3.00)
Health Plan Offer as Multiple of QPA	1.25	(0.68)	1.34	(0.89)	1.31	(0.83)
Winning Offer as Multiple of QPA	2.12	(1.29)	3.83	(3.18)	3.33	(2.87)

Excluding Negative Winning Values and Outliers; Standard Deviations in Parentheses

© 2025 Duffy EL and Garmon C. *JAMA Network Open*.

eTable 3. Rotary Activation Dispute Means and Counts by PE and Non-PE (Nonmonetary File)

	Private Equity					
	Non-PE		PE		Total	
	n	(%)	n	(%)	n	(%)
Dispute Lines	1,981		2,954		4,935	
Health PLan Offer > QPA						
No	1,091	55.1%	1,270	43.0%	2,361	47.8%
Yes	890	44.9%	1,684	57.0%	2,574	52.2%
Outcome						
Health Plan Wins	262	13.2%	382	12.9%	644	13.0%
Provider Wins	1,715	86.6%	2,572	87.1%	4,287	86.9%
Split Decision	4	0.2%	0	0.0%	4	0.1%
	mean	(SD)	mean	(SD)	mean	(SD)
Provider Offer as Multiple of QPA	2.16	(2.05)	3.71	(5.25)	3.09	(4.34)
Health Plan Offer as Multiple of QPA	1.10	(1.03)	1.62	(5.37)	1.40	(4.16)
Winning Offer as Multiple of QPA	2.04	(2.02)	3.47	(5.19)	2.89	(4.27)

Excluding Negative Winning Values and Outliers; Standard Deviations in Parentheses

© 2025 Duffy EL and Garmon C. *JAMA Network Open*.

eTable 4. Characteristics of Disputes for Air Transport Activation by Quarter of 2023 (Nonmonetary File)

	2023 Quarters							
	1		2		3		4	
	n	%	n	%	n	%	n	%
Dispute Lines	1,250		1,646		1,612		1,170	
Health Plan Offer > QPA								
No	828	66.2%	824	50.1%	562	34.9%	478	40.9%
Yes	422	33.8%	822	49.9%	1,050	65.1%	692	59.1%
Outcome								
Health Plan Wins	114	9.1%	259	15.7%	196	12.2%	200	17.1%
Provider Wins	1,136	90.9%	1,385	84.1%	1,414	87.7%	970	82.9%
Split Decision	0	0.0%	2	0.1%	2	0.1%	0	0.0%
	mean	(SD)	mean	(SD)	mean	(SD)	mean	(SD)
Provider Offer as Multiple of QPA	3.01	(4.45)	3.10	(2.92)	3.44	(5.74)	3.10	(2.60)
Health Plan Offer as Multiple of QPA	1.33	(4.25)	1.43	(5.21)	1.40	(3.15)	1.40	(1.06)
Winning Offer as Multiple of QPA	2.87	(4.39)	2.81	(2.70)	3.25	(5.70)	2.83	(2.57)
Days to Make Determination	55.08	(24.70)	64.77	(46.81)	91.43	(59.29)	136.42	(67.55)

Note: Analysis of non-monetary file; Excluding Negative Winning Values and Outliers

© 2025 Duffy EL and Garmon C. *JAMA Network Open*.

eTable 5. Fixed Wing Activation Dispute Means and Counts by Quarter (Nonmonetary File)

	2023 Quarters							
	1		2		3		4	
	n	%	n	%	n	%	n	%
Dispute Lines	130		226		230		157	
Health Plan Offer > QPA								
No	82	63.1%	100	44.2%	84	36.5%	65	41.4%
Yes	48	36.9%	126	55.8%	146	63.5%	92	58.6%
Outcome								
Health Plan Wins	20	15.4%	39	17.3%	32	13.9%	34	21.7%
Provider Wins	110	84.6%	187	82.7%	198	86.1%	123	78.3%
	mean	(SD)	mean	(SD)	mean	(SD)	mean	(SD)
Provider Offer as Multiple of QPA	3.36	(2.75)	4.20	(3.26)	3.66	(2.54)	3.52	(3.36)
Health Plan Offer as Multiple of QPA	1.23	(0.83)	1.24	(0.77)	1.34	(0.89)	1.45	(0.81)
Winning Offer as Multiple of QPA	2.99	(2.34)	3.57	(3.08)	3.42	(2.57)	3.12	(3.30)
Length of Time to Make Determination	49.25	(20.62)	57.43	(40.87)	76.05	(53.58)	131.89	(65.35)

Excluding Negative Winning Values and Outliers

© 2025 Duffy EL and Garmon C. *JAMA Network Open*.

eTable 6. Rotary Activation Dispute Means and Counts by Quarter (Nonmonetary File)

	2023 Quarter							
	1		2		3		4	
	n	%	n	%	n	%	n	%
Dispute Lines	1,120		1,420		1,382		1,013	
Health Plan Offer > QPA								
No	746	66.6%	724	51.0%	478	34.6%	413	40.8%
Yes	374	33.4%	696	49.0%	904	65.4%	600	59.2%
Outcome								
Health Plan Wins	94	8.4%	220	15.5%	164	11.9%	166	16.4%
Provider Wins	1026	91.6%	1198	84.4%	1216	88.0%	847	83.6%
Split Decision	0	0.0%	2	0.1%	2	0.1%	0	0.0%
	mean	(SD)	mean	(SD)	mean	(SD)	mean	(SD)
Provider Offer as Multiple of QPA	2.97	(4.60)	2.93	(2.83)	3.40	(6.10)	3.04	(2.46)
Health Plan Offer as Multiple of QPA	1.34	(4.49)	1.46	(5.60)	1.41	(3.39)	1.40	(1.09)
Winning Offer as Multiple of QPA	2.85	(4.57)	2.68	(2.62)	3.22	(6.07)	2.78	(2.44)
Length of Time to Make Determination	55.75	(25.06)	65.94	(47.60)	93.98	(59.82)	137.12	(67.89)

Excluding Negative Winning Values and Outliers

© 2025 Duffy EL and Garmon C. *JAMA Network Open*.

eTable 7. Masking by Type of Service (Monetary File)

	Air Ambulance Activation	Air Ambulance Mileage	Type of Service Emergency Services	Other	Total
Dispute Lines	5,983	5,838	165,024	168,481	345,326
Level of Masking					
Not masked	2,897	2,678	140,232	108,999	254,806
	48.42%	45.87%	84.98%	64.70%	73.79%
Only QPA masked	72	111	11,541	4,658	16,382
	1.20%	1.90%	6.99%	2.76%	4.74%
Only winning offer masked	88	70	2,763	4,292	7,213
	1.47%	1.20%	1.67%	2.55%	2.09%
Both QPA and winning offer masked	2,926	2,979	10,488	50,532	66,925
	48.91%	51.03%	6.36%	29.99%	19.38%

© 2025 Duffy EL and Garmon C. *JAMA Network Open*.
