# Supplementary material for: No Surprises Act Independent Dispute Resolution Outcomes for Air Ambulances
Source: JAMA Netw Open. 2025 Mar 3;8(3):e2462404. doi: 10.1001/jamanetworkopen.2024.62404 (PMC11877184; doi:10.1001/jamanetworkopen.2024.62404)
Supplement: Supplement 2. — Data Sharing Statement [file jamanetwopen-e2462404-s002.pdf]

Data Sharing Statement

Duffy. No Surprises Act Independent Dispute Resolution Outcomes for Air Ambulances. *JAMA Netw Open*. Published March 03, 2025. doi:10.1001/jamanetworkopen.2024.62404

Data
Data available: No

Additional Information
Explanation for why data not available: These data are already publicly available online via CMS.
